# Case Report: Fremitus Nystagmus in Superior Canal Dehiscence Syndrome

**DOI:** 10.3389/fneur.2022.844687

**Published:** 2022-05-09

**Authors:** Miranda Morrison, Athanasia Korda, Franca Wagner, Marco Domenico Caversaccio, Georgios Mantokoudis

**Affiliations:** ^1^Department of Otorhinolaryngology, Head and Neck Surgery, Inselspital, University Hospital Bern and the University of Bern, Bern, Switzerland; ^2^University Institute of Diagnostic and Interventional Neuroradiology, Inselspital, University Hospital Bern and the University of Bern, Bern, Switzerland

**Keywords:** fremitus, superior canal dehiscence, vertigo, case report, neuro-otology, rare case, atypical features, neurological conditions

## Abstract

Superior canal dehiscence syndrome (SCDS) is a structural bony defect of the roof of the superior semi-circular canal into the middle cranial fossa and is responsible for the creation of a third window, which alters the dynamics of the inner ear. During humming, vibratory waves entering the vestibulum and cochlea are re-routed through the dehiscence, leading to stimulation of the otolithic and ampullary vestibular organs. This is responsible for the torsional-vertical nystagmus known as “fremitus nystagmus”. In this case report, we video-document a rare case of fremitus nystagmus and its resolution after plugging of the superior semi-circular canal.

## Introduction

First described by Minor in 1998 (1), superior semicircular canal (SCC) dehiscence is a structural bony defect of the roof of the SCC, causing the canal to be exposed to the middle cranial fossa (2). This structural abnormality results in a third window, altering the fluid dynamics of the inner ear, thus leading to hearing and balance disturbances. Typically, patients describe symptoms such as hyperacusis, aural fullness, pulsatile tinnitus, and autophonia (heightened awareness of sounds in provenance from their own body, e.g., eye movements in the eye socket, breathing, heartbeat, etc) (3). Occasionally, they suffer from episodes of vertigo; this can manifest itself as either chronic dizziness-instability or repetitive episodes of pressure/sound or straining-induced rotatory vertigo (4). The Tullio phenomenon, where a patient suffers from an acute vertigo attack when exposed to loud noises, is well-documented (5). This is thought to be due to the “shunt effect” of the third window that creates a traveling wave that stimulates the cupula (2, 6). Additionally, there is a loss of energy through the third window, leading to loss of acoustic energy (7) and low-frequency conductive hearing loss with decreased bone conduction thresholds due to changes in impedance of the cochlea on the side of the scala vestibuli. This can be mistaken for otosclerosis. This dynamic alteration is, thus, thought to be responsible for the so-called “fremitus nystagmus”, vertical-torsional nystagmus provoked when a subject emits a humming noise between 200 and 2,000 Hz (2). “Fremitus” refers to the transmission of sound vibration throughout the body and is commonly used in the description of certain pulmonary and cardiac pathologies. In “fremitus nystagmus”, this vibration produced by humming travels up to the inner ear where it stimulates the cupula of the superior semicircular canal; thus, the resulting nystagmus corresponds to the plane of the stimulated canal. This phenomenon was first video-documented by Gürkov et al. whose 2017 article describes 4 cases of SCDS, out of which two patients had “fremitus nystagmus”. The prevalence of this condition still remains unknown but is estimated to be anywhere between 0.4 and 9% (2, 8). SCDS remains challenging to diagnose, with a wide variance in severity of symptomatic presentation (3), and with only a few cases suffering from disabling symptoms. Ocular Vestibular Evoked Myogenic Potential (oVEMPs) and high-resolution CT scans are the most reliable methods for their detection (2, 9). Different surgical techniques have been described such as SCC re-surfacing (10), capping (11), plugging (12), and simple reinforcement of the round and oval window (11, 13, 14) and are reserved only for the most severe of cases. Currently, there are three different surgical approaches, the middle fossa and transmastoidal approaches are the most common and the transcanal (transmeatal) or endaural approach (3). Here, we present the video-documented resolution of fremitus nystagmus after transmastoidal surgical plugging of semicircular canal dehiscence.

## Case Description

We examined the case of a 66-year-old male patient, who was addressed to our clinic with a hearing and balance disorder. The patient described disturbing left-sided hyperacusis with tinnitus and autophonia. He experienced oscillopsia while running but had never presented with the Tullio phenomenon. He also experienced a vertical sensation of self motion while humming. For the past 20 years, he has been incapable of wearing any shoes other than his running shoes, whose thick soles cushioned the vibrations induced by his steps, thus decreasing his symptoms. It was difficult for him to talk aloud and when participating in normal conversations he often found himself having to whisper. There was no family history of hearing impairment.

**Figure 1:**
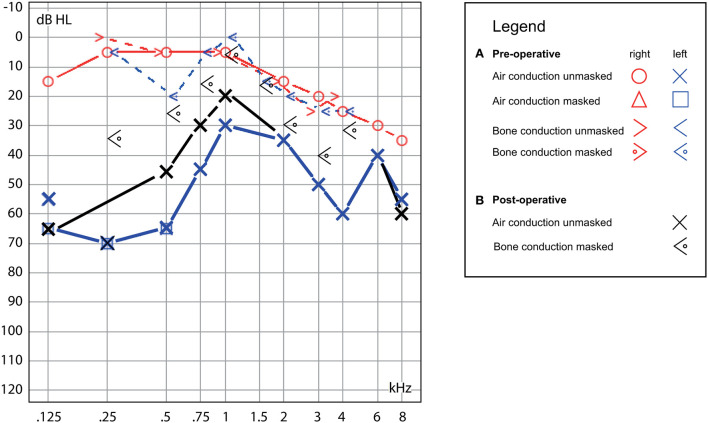
Hearing test. **(A)** Preoperative, demonstrating left-sided low-frequency conductive hearing loss with mild high-frequency hearing loss on the contralateral ear (blue/red traces) and **(B)** postoperative, with the persistence of similar conductive hearing loss on the left (black traces).

**Figure 2:**
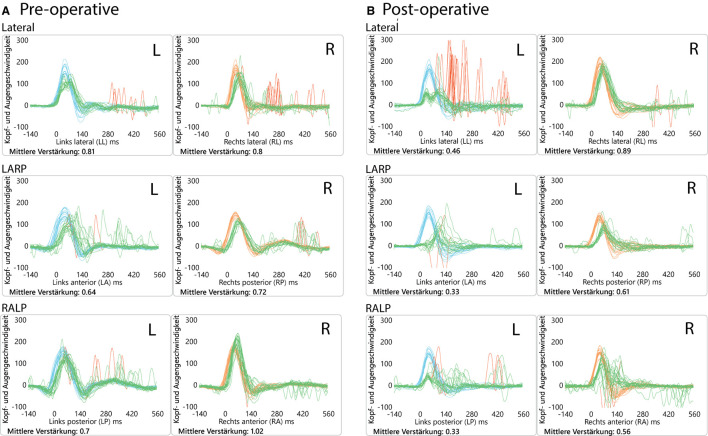
Video head impulse test (vHIT) results. **(A)** Preoperatively, showing anterior canal hypofunction and **(B)** postoperatively (2 months) showing diminished VOR gain values on the left side (operated side) for all 3 canals. This initial left-sided postoperative hypofunction fully recovered over time.

**Figure 3:**
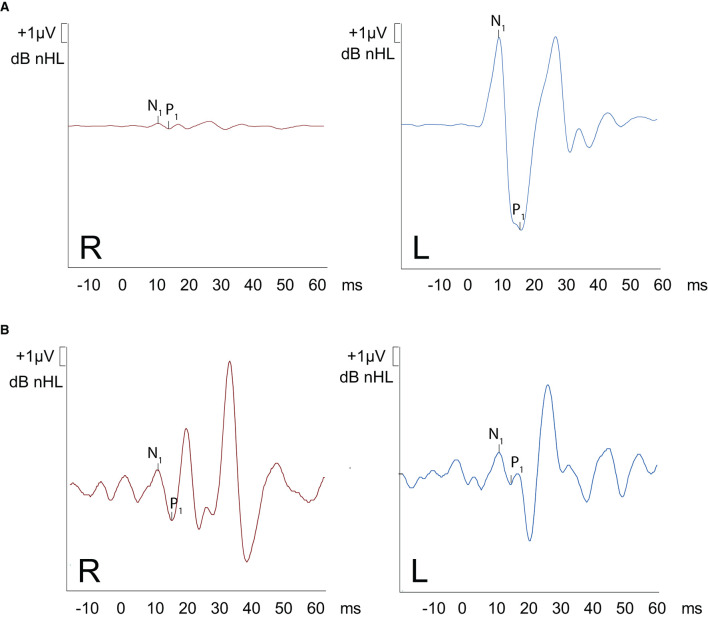
oVEMPS performed **(A)** preoperatively and **(B)** postoperatively. **(A)** A substantial increase in trace amplitude (based on normative data) is present on the left ear, with normal traces on the right ear confirming the presence of left-sided SCC-Dehiscence. The peak-to-peak asymmetry was 94%. **(B)** Postoperative findings demonstrate symmetrical results on both ears (amplitudes restored back to normal values).

**Figure 4:**
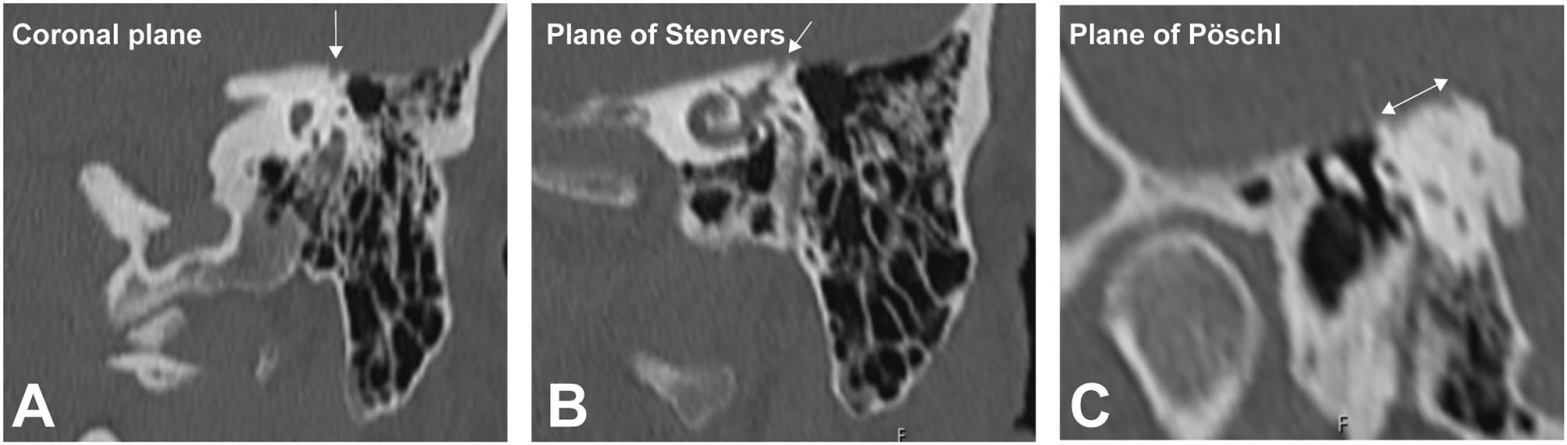
High-resolution temporal bone imaging CT (slice thickness 0.6 mm). The semicircular canal (SCC) dehiscence is indicated by the white arrows in the **(A)** coronal plane, **(B)** plane of Stenvers, and **(C)** plane of Pöschl.

The patient had a previous unclear history of “round and oval window reinforcement” as a treatment for his hyperacusis (15), undertaken 20 years prior, which had worsened his hearing and led to disturbing tinnitus. In the clinical workup, we performed an otoscopy and tympanometry, which were normal. The tuning fork examination showed left-sided lateralization on the Weber test combined with a negative left-sided Rinne test. The clinical vestibular workup was normal with the exception of a mixed horizontal (leftbeat)/vertical (downbeat) and torsional nystagmus with the top pole rotating to the left ear during sustained humming. The onset of the nystagmus was immediate (no latency), increasing in amplitude and speed (crescendo) until discontinuation of stimulus, at which point the nystagmus ceased completely. The fistula sign was negative. On the hearing test, there was left-sided low-frequency conductive hearing loss (Figure 1), which was either due to the presence of middle ear scar tissue linked to his prior middle ear surgery or conductive hearing loss associated with the SCC dehiscence (16). The preoperative vHIT demonstrated hypofunction of the left anterior canal with a gain of 0.64 and with normal gains bilaterally in all other canals (Figure 2). On the oVEMPS, amplitudes were elevated on the left hand side (Figure 3) with significant asymmetry (94%) of the peak-to-peak amplitude. On the Cervical Vestibular Evoked Myogenic Potential (cVEMPs), there were no responses on the left side due to significant conductive hearing loss. A neuroradiological workup was undertaken in our institution; on the thin cut high-resolution temporal bone CT with a slice thickness of 0.6 mm, there was a bony discontinuity along the roof of the left superior SCC with a dehiscence measuring 5.4 mm (Figure 4).

**Figure 5:**
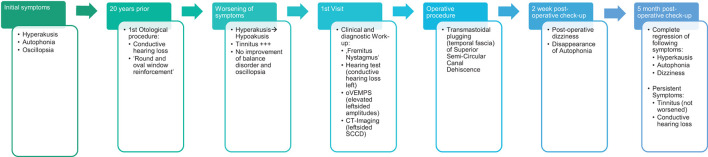
Case report Timeline.

The patient was scheduled for left transmastoidal plugging of the superior SCC dehiscence (Figure 5). We skeletonized and blue-lined the left superior SCC at the ampulated and non-ampulated end without identifying the actual dehiscence. The canal was then opened with an underwater endoscopic technique (14) at either end (ampullary and non-ampullary) and plugged using wet temporal fascia and covered with bone dust, thus leading to its complete occlusion. The patient remained hospitalized for 3 days, during which he received corticosteroids and anti-emetics because of postoperative nausea and vertigo, which resolved over time (17).

A postoperative checkup was performed after 2 weeks to remove the operative gauze from the ear and after 2 and 5 months. The patient no longer suffered from autophonia, thus enabling him to give speeches/sermons at his local church. He was able to take up sport again with the disappearance of the running-induced oscillopsia. The only residual symptoms were the pre-existing tinnitus and conductive hearing loss, which, after the plugging of the anterior canal, neither improved nor worsened (Figure 1). The video head impulse test (vHIT) showed on the left side (operated side) normal Vestibulo-Ocular reflex (VOR) gain values for the lateral semicircular canal but pathological gains for the posterior and anterior canals (Figure 2). oVEMPs were symmetrical (reduced to 20% asymmetry) in their responses with normal amplitudes. The fremitus nystagmus that had initially been clearly visible during humming completely vanished postoperatively.

## Discussion

The fremitus nystagmus displayed by this patient with superior SCC dehiscence is a hallmark of change in the biomechanics of the inner ear. It is thought to be due to the “third window” effect where sound waves, upon entering the inner ear, are shunted directly through the SCC dehiscence, thus erroneously stimulating otolithic organs of the vestibulum and the ampulla of the superior semicircular canal (2, 18–20).

Using chinchilla models, Kaski et al. recreated artificial SCC dehiscence through SCC fenestration. A fistula test was then performed where the air was insufflated into the ear, producing an ampullo-fugal and -petal motion of endolymph simulating this “shunt effect.” The induced eye movements were in the same plane as the stimulated canal (5), which is also known from Ewald's first law.

This was further explored by Aw et al. who studied 18 patients with SCC dehiscence (superior and posterior) and recorded their eye movements using scleral search coils during vibratory stimulation of the mastoid with a bone oscillator. They also found that upon stimulation, patients with superior SCC dehiscence displayed eye movements (nystagmus) that followed referenced known semicircular canal planes (19).

Although the pathophysiology of SCC dehiscence is well-documented, the occurrence of fremitus nystagmus is not. To our knowledge, there is currently only one article documenting fremitus nystagmus; Gürkov et al. reported 4 cases of SCC dehiscence, out of which two presented with fremitus nystagmus (2). It is described as self-generated vertical torsional nystagmus due to bone-conducted vibration (21). The expected direction of the induced nystagmus would be in the plane of the affected canal following Ewald's first law (22). Nystagmus is often accompanied by a contralateral tilt of the head and the subjective visual vertical (2, 23). However, with our patient, the direction of the fast phase of nystagmus was predominantly torsional, with the top pole rotating toward the left ear with a small vertical-downward component measurable on video-oculography (2). These movements can be explained by the excitatory ampullofugal displacement of the cupula of the superior SCC (2, 24). A horizontal left beating component to the nystagmus was occasionally visible and could potentially be attributed to utricular stimulation (25, 26). The frequency of humming was around the 230–280Hz range, which is close to optimal frequency response (21).

## Limitations

Fremitus nystagmus remains a rare and atypical finding in patients with a third window: emphasis of it as a clinical sign could divert the readers' attention from the more common findings in SCDS (hyperacusis, aural fullness, autophonia, vertigo, etc). Many of these signs and symptoms are similar to those of other inner ear pathologies (e.g., Morbus Ménière, otosclerosis), making SCDS sometimes difficult to diagnose. Additionally, it should be mentioned that this case of SCDS had a previous history of middle ear surgery. Theoretically, it cannot be completely excluded that the “fremitus nystagmus” in this case reflects some other (surgically induced) dynamic phenomenon that is still unaccounted for.

## Conclusion

Fremitus nystagmus is an eye movement occurring in patients with SCDS during sustained humming. It is predominantly torsional “nystagmus,” with the top pole of the eye rotating toward the affected ear. Superior SCC dehiscence should be diagnosed according to current diagnostic criteria (4) including thorough clinical examination, hearing test, oVEMPs, and temporal bone imaging (high-resolution CT). Fremitus nystagmus resolves after transmastoidal surgical plugging of the affected superior SCC.

## Data Availability Statement

The original contributions presented in the study are included in the article/Supplementary Material, further inquiries can be directed to the corresponding author.

## Ethics Statement

Written informed consent was obtained from the individual(s) for the publication of any potentially identifiable images or data included in this article.

## Author Contributions

MM is responsible for case writing and literature indexing. GM and AK are responsible for case summary and full text guidance. FW was responsible for radiological image analysis. All authors contributed to the article and approved the submitted version.

## Funding

GM was supported by the Swiss National Science Foundation (#320030_173081).

## Conflict of Interest

The authors declare that the research was conducted in the absence of any commercial or financial relationships that could be construed as a potential conflict of interest.

## Publisher's Note

All claims expressed in this article are solely those of the authors and do not necessarily represent those of their affiliated organizations, or those of the publisher, the editors and the reviewers. Any product that may be evaluated in this article, or claim that may be made by its manufacturer, is not guaranteed or endorsed by the publisher.

## Supplementary Material

Supplementary Video 1 **(A)** Preoperative “fremitus nystagmus”, **(B)** semicircular Canal Dehiscence (SCCD) plugging, and **(C)** complete postoperative resolution of the nystagmus.
